# (*S*)-(−)-6-(4-Bromo­phen­yl)-2,3,5,6-tetra­hydro­thia­zolo[2,3-*b*]imidazolium hydrogen oxalate

**DOI:** 10.1107/S1600536808029085

**Published:** 2008-09-17

**Authors:** Thomas Minor, Maksymilian Chruszcz

**Affiliations:** a Department of Molecular Physiology and Biological Physics, University of Virginia, 1340 Jefferson Park Avenue, Charlottesville, VA 22908, USA

## Abstract

The structure of the title compound, C_11_H_12_BrN_2_S^+^·C_2_HO_4_^−^ (common name 6-bromo­levamisole hydrogen oxalate), is stabilized mainly by hydrogen bonds. Hydrogen oxalate anions form parallel coplanar chains *via* O—H⋯O hydrogen bonds, while there are N—H⋯O hydrogen-bonding inter­actions between the 6-bromo­levamisole cations and oxalate anions. Both five-membered rings from the 6-bromo­levamisole mol­ecule have a twist conformation. The mol­ecule has an extended conformation, with the 4-bromo­phenyl substituent positioned equatorially with N—C—C—C and C—C—C—C torsion angles of 39.8 (3) and 100.4 (3)°, respectively.

## Related literature

For background information, see: Denier *et al.* (2002 ▶); Lee *et al.* (1975 ▶); Luo *et al.* (2000 ▶).
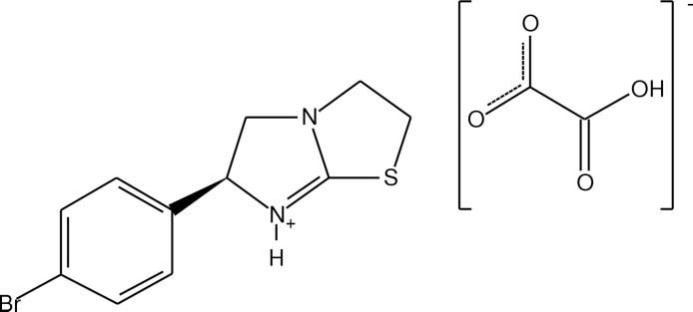


## Experimental

### 

#### Crystal data

C_11_H_12_BrN_2_S^+^·C_2_HO_4_^−^
*M* *_r_* = 373.22
Orthorhombic, 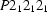

*a* = 5.615 (1) Å
*b* = 8.256 (1) Å
*c* = 32.539 (1) Å
*V* = 1508.4 (3) Å^3^
*Z* = 4
Mo *K*α radiation
μ = 2.88 mm^−1^
*T* = 89 (2) K
0.50 × 0.03 × 0.03 mm

#### Data collection

Rigaku R-AXIS RAPID diffractometer
Absorption correction: multi-scan (Otwinowski *et al.*, 2003 ▶) *T*_min_ = 0.90, *T*_max_ = 0.92
39658 measured reflections
4061 independent reflections
3438 reflections with *I* > 2σ(*I*)
*R*_int_ = 0.081

#### Refinement

*R*[*F* ^2^ > 2σ(*F* ^2^)] = 0.032
*wR*(*F* ^2^) = 0.074
*S* = 1.10
4061 reflections
230 parameters
H atoms treated by a mixture of independent and constrained refinement
Δρ_max_ = 0.67 e Å^−3^
Δρ_min_ = −0.51 e Å^−3^
Absolute structure: Flack (1983 ▶), 1863 Friedel pairs
Flack parameter: −0.018 (7)

### 

Data collection: *HKL-2000* (Otwinowski & Minor, 1997 ▶); cell refinement: *HKL-2000*; data reduction: *HKL-2000*; program(s) used to solve structure: *SHELXS97* (Sheldrick, 2008 ▶) and *HKL-3000SM* (Minor *et al.*, 2006 ▶); program(s) used to refine structure: *SHELXL97* (Sheldrick, 2008 ▶) and *HKL-3000SM*; molecular graphics: *HKL-3000SM*, *Mercury* (Macrae *et al.*, 2006 ▶), *ORTEPIII* (Burnett & Johnson, 1996 ▶) and *ORTEP-3* (Farrugia, 1997 ▶); software used to prepare material for publication: *HKL-3000SM*.

## Supplementary Material

Crystal structure: contains datablocks I, global. DOI: 10.1107/S1600536808029085/fl2215sup1.cif

Structure factors: contains datablocks I. DOI: 10.1107/S1600536808029085/fl2215Isup2.hkl

Additional supplementary materials:  crystallographic information; 3D view; checkCIF report

## Figures and Tables

**Table 1:** Hydrogen-bond geometry (Å, °)

*D*—H⋯*A*	*D*—H	H⋯*A*	*D*⋯*A*	*D*—H⋯*A*
O1—H1*O*⋯O3^i^	1.03 (5)	1.48 (5)	2.483 (2)	164 (4)
N2—H1*N*⋯O4	0.83 (4)	1.95 (4)	2.753 (3)	163 (4)
N2—H1*N*⋯O1	0.83 (4)	2.37 (4)	2.879 (3)	120 (3)

Symmetry code: (i) 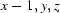.

**Fig. 1.**
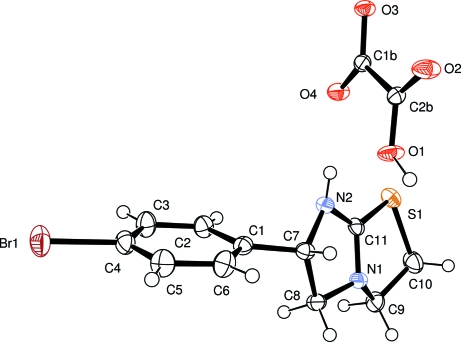
An asymmetric unit of the title compound. Displacement ellipsoids are drawn at the 50% probability level, while hydrogen atoms are drawn as spheres of an arbitrary radius.

**Fig. 2.**
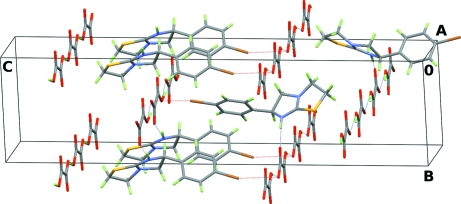
A packing diagram with hydrogen bonds marked with blue, dashed lines. Short contacts between Br···O3 (-1/2 + x, 1/2 - y, 2 - z) are marked with red, dashed lines.

## supplementary crystallographic information

### Comment

6-Bromolevamisole (Fig. 1) is a salt of a strong activator of cystic fibrosis conductance regulator (CTFR) chloride channels, including those in human airway epithelial cells. It shows a strong reduction in activity of Protein Phosphatases 2C and 2A, two of the most likely candidates for being a CTFR phosphatase (Luo *et al.*, 2000). It has also been shown to inhibit alkaline phosphatases, including being an uncompetitive inhibitor of an alkaline phosphatase involved in sarcoma (Lee *et al.*, 1975). Furthermore, since inhibitors affect alkaline phosphatase from the white blood cells of mothers of fetuses with Down's syndrome differently, the cation could be involved in screening for it (Denier *et al.*, 2002).

Packing (Fig. 2) is stabilized by hydrogen bonds (Table 1). The oxalate ions form parallel, coplanar, one-dimensional chains *via* O—H···O hydrogen bonds, with each link in the chain having an N—H···O hydrogen bond from the deprotonated oxygen to the protonated nitrogen (N2) of the 6-bromolevamisole. The bromine also forms a short contact (3.111 Å) with the O3 (-1/2 + *x*, 1/2 - *y*, 2 - *z*) atom.

### Experimental

6-Bromolevamisole oxalate was purchased from Sigma, and dissolved in a mixture of 1-butanol and DMSO in a 1:1 ratio. A single crystal suitable for X-ray diffraction study was obtained by slow evaporation at room temperature.

### Refinement

Hydrogen atoms attached to C7, C8, and C9 were placed in ideal positions, and refined using a riding-model approximation with C—H bond lengths of 0.98 Å in the case of C7 and 0.97 Å in the cases of C8 and C9. All other hydrogen atoms were located in a difference density Fourier map and refined with isotropic displacement parameters.

### Crystal data

**Table:**

C_11_H_12_BrN_2_S^+^·C_2_HO_4_^−^	*D*_x_ = 1.643 Mg m^−^^3^
*M**_r_* = 373.22	Melting point: 465 K
Orthorhombic, *P*2_1_2_1_2_1_	Mo *K*α radiation, λ = 0.71074 Å
Hall symbol: P 2ac 2ab	Cell parameters from 39658 reflections
*a* = 5.615 (1) Å	θ = 2.6–29.1°
*b* = 8.256 (1) Å	µ = 2.88 mm^−^^1^
*c* = 32.539 (1) Å	*T* = 89 K
*V* = 1508.4 (3) Å^3^	Needle, colourless
*Z* = 4	0.50 × 0.03 × 0.03 mm
*F*(000) = 752	

### Data collection

**Table:**

Rigaku R-AXIS RAPID diffractometer	4061 independent reflections
Radiation source: fine-focus sealed tube	3438 reflections with *I* > 2σ(*I*)
graphite	*R*_int_ = 0.081
Detector resolution: 10 pixels mm^-1^	θ_max_ = 29.1°, θ_min_ = 2.6°
ω scans with χ offset	*h* = −7→7
Absorption correction: multi-scan (Otwinowski *et al.*, 2003)	*k* = −11→11
*T*_min_ = 0.90, *T*_max_ = 0.92	*l* = −44→44
39658 measured reflections	

### Refinement

**Table:**

Refinement on *F*^2^	Secondary atom site location: difference Fourier map
Least-squares matrix: full	Hydrogen site location: inferred from neighbouring sites
*R*[*F*^2^ > 2σ(*F*^2^)] = 0.032	H atoms treated by a mixture of independent and constrained refinement
*wR*(*F*^2^) = 0.074	*w* = 1/[σ^2^(*F*_o_^2^) + (0.0326*P*)^2^ + 0.8007*P*] where *P* = (*F*_o_^2^ + 2*F*_c_^2^)/3
*S* = 1.10	(Δ/σ)_max_ = 0.001
4061 reflections	Δρ_max_ = 0.67 e Å^−^^3^
230 parameters	Δρ_min_ = −0.51 e Å^−^^3^
0 restraints	Absolute structure: Flack (1983), 1686 Friedel pairs?
Primary atom site location: structure-invariant direct methods	Flack parameter: −0.018 (7)

### Special details

**Table:**

Geometry. All e.s.d.'s (except the e.s.d. in the dihedral angle between two l.s. planes) are estimated using the full covariance matrix. The cell e.s.d.'s are taken into account individually in the estimation of e.s.d.'s in distances, angles and torsion angles; correlations between e.s.d.'s in cell parameters are only used when they are defined by crystal symmetry. An approximate (isotropic) treatment of cell e.s.d.'s is used for estimating e.s.d.'s involving l.s. planes.

### Fractional atomic coordinates and isotropic or equivalent isotropic displacement parameters (Å^2^)

**Table:**

	*x*	*y*	*z*	*U*_iso_*/*U*_eq_	
Br1	0.32101 (6)	0.56744 (4)	1.059826 (8)	0.03817 (9)	
S1	0.35196 (13)	0.49266 (9)	0.76858 (2)	0.02854 (14)	
N2	0.2692 (4)	0.4064 (3)	0.84952 (6)	0.0204 (4)	
C11	0.2064 (4)	0.4764 (3)	0.81524 (7)	0.0196 (5)	
C7	0.0668 (4)	0.4192 (3)	0.87900 (7)	0.0198 (5)	
H7	−0.0267	0.3190	0.8781	0.024*	
N1	−0.0024 (4)	0.5521 (3)	0.81664 (6)	0.0201 (4)	
C8	−0.0808 (4)	0.5585 (3)	0.85977 (7)	0.0211 (5)	
H8A	−0.2505	0.5387	0.8622	0.025*	
H8B	−0.0427	0.6620	0.8723	0.025*	
C9	−0.0363 (6)	0.6744 (4)	0.78469 (8)	0.0261 (6)	
C1	0.1407 (4)	0.4519 (3)	0.92285 (7)	0.0208 (5)	
C2	0.3255 (5)	0.5587 (3)	0.93230 (7)	0.0247 (5)	
C10	0.0970 (6)	0.6059 (4)	0.74766 (9)	0.0329 (7)	
C3	0.3820 (5)	0.5913 (4)	0.97307 (9)	0.0307 (6)	
C5	0.0649 (5)	0.4154 (4)	0.99535 (9)	0.0313 (6)	
C6	0.0123 (6)	0.3807 (4)	0.95449 (9)	0.0288 (6)	
C4	0.2489 (5)	0.5198 (3)	1.00383 (8)	0.0272 (6)	
O3	0.8027 (3)	−0.0585 (2)	0.84464 (5)	0.0197 (3)	
O4	0.6388 (3)	0.1887 (2)	0.84329 (6)	0.0238 (4)	
C1B	0.6295 (4)	0.0378 (3)	0.84479 (7)	0.0172 (5)	
C2B	0.3822 (4)	−0.0442 (3)	0.84594 (7)	0.0186 (5)	
O1	0.2089 (3)	0.0602 (2)	0.84707 (6)	0.0279 (4)	
O2	0.3598 (3)	−0.1898 (2)	0.84549 (7)	0.0320 (5)	
H2	0.405 (6)	0.611 (4)	0.9106 (11)	0.040 (10)*	
H5	−0.032 (7)	0.372 (5)	1.0134 (12)	0.051 (11)*	
H10B	0.158 (8)	0.694 (5)	0.7302 (12)	0.058 (11)*	
H9A	−0.201 (7)	0.684 (4)	0.7791 (9)	0.027 (8)*	
H3	0.500 (7)	0.655 (4)	0.9791 (10)	0.037 (9)*	
H9B	0.031 (6)	0.780 (4)	0.7948 (9)	0.027 (8)*	
H10A	0.009 (7)	0.530 (5)	0.7315 (12)	0.052 (11)*	
H6	−0.104 (7)	0.305 (5)	0.9487 (10)	0.045 (10)*	
H1O	0.042 (9)	0.012 (5)	0.8512 (14)	0.076 (15)*	
H1N	0.361 (7)	0.327 (4)	0.8501 (11)	0.044 (10)*	

### Atomic displacement parameters (Å^2^)

**Table:**

	*U*^11^	*U*^22^	*U*^33^	*U*^12^	*U*^13^	*U*^23^
Br1	0.0587 (2)	0.03403 (15)	0.02182 (11)	0.00178 (15)	−0.00718 (13)	−0.00204 (11)
S1	0.0306 (3)	0.0314 (3)	0.0237 (3)	0.0028 (3)	0.0062 (3)	−0.0047 (2)
N2	0.0192 (10)	0.0191 (11)	0.0230 (9)	0.0027 (8)	−0.0002 (8)	−0.0023 (8)
C11	0.0207 (11)	0.0133 (12)	0.0248 (11)	−0.0011 (9)	−0.0004 (9)	−0.0053 (8)
C7	0.0188 (11)	0.0176 (12)	0.0230 (11)	0.0011 (10)	0.0009 (9)	−0.0003 (9)
N1	0.0192 (9)	0.0205 (11)	0.0206 (9)	0.0028 (9)	−0.0001 (7)	−0.0001 (8)
C8	0.0186 (11)	0.0235 (12)	0.0212 (10)	0.0031 (11)	0.0009 (8)	0.0011 (10)
C9	0.0315 (16)	0.0259 (14)	0.0209 (12)	0.0032 (12)	−0.0035 (11)	0.0018 (10)
C1	0.0212 (12)	0.0186 (12)	0.0225 (10)	0.0015 (10)	−0.0018 (9)	0.0009 (9)
C2	0.0248 (12)	0.0240 (12)	0.0254 (11)	−0.0056 (12)	0.0014 (10)	−0.0017 (9)
C10	0.0430 (18)	0.0332 (16)	0.0225 (12)	0.0072 (13)	−0.0009 (12)	−0.0022 (11)
C3	0.0328 (16)	0.0310 (16)	0.0282 (13)	−0.0079 (12)	−0.0028 (11)	−0.0046 (11)
C5	0.0358 (15)	0.0316 (16)	0.0263 (12)	−0.0052 (13)	0.0022 (11)	0.0044 (12)
C6	0.0315 (15)	0.0262 (14)	0.0285 (13)	−0.0081 (12)	−0.0030 (11)	0.0025 (11)
C4	0.0369 (14)	0.0251 (13)	0.0195 (11)	0.0047 (11)	−0.0038 (10)	0.0006 (9)
O3	0.0130 (7)	0.0178 (7)	0.0285 (8)	0.0007 (7)	0.0008 (6)	−0.0009 (7)
O4	0.0174 (9)	0.0172 (8)	0.0368 (9)	−0.0001 (7)	−0.0003 (8)	−0.0004 (7)
C1B	0.0153 (10)	0.0211 (13)	0.0153 (9)	0.0003 (9)	0.0006 (8)	−0.0010 (8)
C2B	0.0153 (11)	0.0214 (13)	0.0191 (10)	0.0004 (9)	−0.0011 (8)	−0.0012 (9)
O1	0.0113 (8)	0.0192 (8)	0.0532 (11)	0.0004 (7)	0.0000 (7)	−0.0015 (9)
O2	0.0170 (9)	0.0183 (9)	0.0606 (13)	−0.0004 (7)	0.0001 (9)	−0.0025 (9)

### Geometric parameters (Å, °)

**Table:**

Br1—C4	1.908 (3)	C1—C2	1.396 (4)
S1—C11	1.729 (2)	C2—C3	1.390 (3)
S1—C10	1.840 (3)	C2—H2	0.94 (4)
N2—C11	1.305 (3)	C10—H10B	0.98 (4)
N2—C7	1.491 (3)	C10—H10A	0.96 (4)
N2—H1N	0.83 (4)	C3—C4	1.382 (4)
C11—N1	1.329 (3)	C3—H3	0.87 (4)
C7—C1	1.510 (3)	C5—C4	1.373 (4)
C7—C8	1.549 (3)	C5—C6	1.392 (4)
C7—H7	0.9800	C5—H5	0.88 (4)
N1—C9	1.461 (3)	C6—H6	0.92 (4)
N1—C8	1.472 (3)	O3—C1B	1.257 (3)
C8—H8A	0.9700	O4—C1B	1.248 (3)
C8—H8B	0.9700	C1B—C2B	1.546 (3)
C9—C10	1.527 (4)	C2B—O2	1.208 (3)
C9—H9A	0.95 (4)	C2B—O1	1.300 (3)
C9—H9B	1.00 (3)	O1—H1O	1.03 (5)
C1—C6	1.387 (4)		
			
C11—S1—C10	89.80 (13)	C2—C1—C7	121.7 (2)
C11—N2—C7	108.2 (2)	C3—C2—C1	120.2 (2)
C11—N2—H1N	122 (2)	C3—C2—H2	121 (2)
C7—N2—H1N	121 (3)	C1—C2—H2	118 (2)
N2—C11—N1	114.6 (2)	C9—C10—S1	106.10 (19)
N2—C11—S1	131.1 (2)	C9—C10—H10B	111 (2)
N1—C11—S1	114.23 (18)	S1—C10—H10B	109 (3)
N2—C7—C1	114.3 (2)	C9—C10—H10A	115 (2)
N2—C7—C8	101.55 (19)	S1—C10—H10A	106 (2)
C1—C7—C8	113.3 (2)	H10B—C10—H10A	110 (3)
N2—C7—H7	109.1	C4—C3—C2	119.0 (3)
C1—C7—H7	109.1	C4—C3—H3	121 (2)
C8—C7—H7	109.1	C2—C3—H3	120 (2)
C11—N1—C9	114.5 (2)	C4—C5—C6	118.7 (3)
C11—N1—C8	108.2 (2)	C4—C5—H5	126 (3)
C9—N1—C8	127.9 (2)	C6—C5—H5	115 (3)
N1—C8—C7	101.46 (19)	C1—C6—C5	120.8 (3)
N1—C8—H8A	111.5	C1—C6—H6	120 (2)
C7—C8—H8A	111.5	C5—C6—H6	119 (2)
N1—C8—H8B	111.5	C5—C4—C3	122.0 (3)
C7—C8—H8B	111.5	C5—C4—Br1	118.7 (2)
H8A—C8—H8B	109.3	C3—C4—Br1	119.3 (2)
N1—C9—C10	104.0 (2)	O4—C1B—O3	126.8 (2)
N1—C9—H9A	108.6 (19)	O4—C1B—C2B	118.5 (2)
C10—C9—H9A	110.9 (18)	O3—C1B—C2B	114.69 (19)
N1—C9—H9B	108.4 (17)	O2—C2B—O1	125.6 (2)
C10—C9—H9B	113.1 (18)	O2—C2B—C1B	121.9 (2)
H9A—C9—H9B	111 (3)	O1—C2B—C1B	112.4 (2)
C6—C1—C2	119.3 (2)	C2B—O1—H1O	115 (3)
C6—C1—C7	118.9 (2)		
			
C7—N2—C11—N1	6.2 (3)	N2—C7—C1—C2	39.8 (3)
C7—N2—C11—S1	−175.80 (19)	C8—C7—C1—C2	−75.9 (3)
C10—S1—C11—N2	179.2 (3)	C6—C1—C2—C3	0.9 (4)
C10—S1—C11—N1	−2.8 (2)	C7—C1—C2—C3	177.2 (2)
C11—N2—C7—C1	−141.1 (2)	N1—C9—C10—S1	−32.9 (3)
C11—N2—C7—C8	−18.8 (2)	C11—S1—C10—C9	21.4 (2)
N2—C11—N1—C9	159.8 (2)	C1—C2—C3—C4	−1.6 (4)
S1—C11—N1—C9	−18.5 (3)	C2—C1—C6—C5	0.4 (4)
N2—C11—N1—C8	10.3 (3)	C7—C1—C6—C5	−176.0 (3)
S1—C11—N1—C8	−168.00 (17)	C4—C5—C6—C1	−0.9 (5)
C11—N1—C8—C7	−21.0 (3)	C6—C5—C4—C3	0.2 (4)
C9—N1—C8—C7	−165.2 (2)	C6—C5—C4—Br1	180.0 (2)
N2—C7—C8—N1	23.0 (2)	C2—C3—C4—C5	1.1 (4)
C1—C7—C8—N1	146.1 (2)	C2—C3—C4—Br1	−178.7 (2)
C11—N1—C9—C10	33.8 (3)	O4—C1B—C2B—O2	176.5 (2)
C8—N1—C9—C10	176.1 (2)	O3—C1B—C2B—O2	−2.1 (3)
N2—C7—C1—C6	−143.9 (3)	O4—C1B—C2B—O1	−3.0 (3)
C8—C7—C1—C6	100.4 (3)	O3—C1B—C2B—O1	178.4 (2)

### Hydrogen-bond geometry (Å, °)

**Table:**

*D*—H···*A*	*D*—H	H···*A*	*D*···*A*	*D*—H···*A*
O1—H1O···O3^i^	1.03 (5)	1.48 (5)	2.483 (2)	164 (4)
N2—H1N···O4	0.83 (4)	1.95 (4)	2.753 (3)	163 (4)
N2—H1N···O1	0.83 (4)	2.37 (4)	2.879 (3)	120 (3)

Symmetry codes: (i) *x*−1, *y*, *z*.
